# Comparison of mutation spectra induced by gamma-rays and carbon ion beams

**DOI:** 10.1093/jrr/rrae050

**Published:** 2024-06-28

**Authors:** Yuka Tokuyama, Kanae Mori, Midori Isobe, Hiroaki Terato

**Affiliations:** Analytical Research Center for Experimental Science, Saga University, 5-1-1 Nabeshima, Saga 849-8501, Japan; Analytical Research Center for Experimental Science, Saga University, 5-1-1 Nabeshima, Saga 849-8501, Japan; Advanced Science Research Center, Okayama University, 2-5-1 Shikata-cho, Kita-ku, Okayama 700-8558, Japan; Advanced Science Research Center, Okayama University, 2-5-1 Shikata-cho, Kita-ku, Okayama 700-8558, Japan

**Keywords:** base damage, mutation, gamma-rays, heavy ion beam

## Abstract

The ionizing radiation with high linear energy transfer (LET), such as a heavy ion beam, induces more serious biological effects than low LET ones, such as gamma- and X-rays. This indicates a difference in the DNA damage produced by low and high LET radiations and their biological effects. We have been studying the differences in DNA damage produced by gamma-rays and carbon ion beams. Therefore, we analyze mutations induced by both ionizing radiations to discuss the differences in their biological effects in this study. pUC19 plasmid DNA was irradiated by carbon ion beams in the solution containing 1M dimethyl sulfoxide to mimic a cellular condition. The irradiated DNA was cloned in competent cells of *Escherichia coli*. The clones harboring some mutations in the region of lacZα were selected, and the sequence alterations were analyzed. A one-deletion mutation is significant in the carbon-irradiated DNA, and the C:G↔T:A transition is minor. On the other hand, the gamma-irradiated DNA shows mainly G:C↔T:A transversion. These results suggest that carbon ion beams produce complex DNA damage, and gamma-rays are prone to single oxidative base damage, such as 8-oxoguanine. Carbon ion beams can also introduce oxidative base damage, and the damage species is 5-hydroxycytosine. This was consistent with our previous results of DNA damage caused by heavy ion beams. We confirmed the causal DNA damage by mass spectrometry for these mutations.

## INTRODUCTION

Ionizing radiation causes severe acute and chronic biological consequences. We know that different kinds of ionizing radiation have different consequences. Heavy ion beams usually produce more severe results than ordinary gamma- and X-rays. This reason is thought to be from the condensed ionization around the tracks of heavy ion beams. The condensed ionization gives the target molecules complex damage. When DNA is the target molecule, this complex damage is called ‘clustered DNA damage’. Clustered DNA damage consists of multiple base lesions and strand breaks in a small and restricted region [1, 2]. Clustered DNA damage is a double-strand break (DSB) if it consists solely of strand breaks and the breaks are located separately on each strand. We previously showed that clustered DNA damage is effectively induced by heavy ion beams [3, 4]. These results from our previous studies are consistent with the results of many other studies [5]. Clustered DNA damage, including DSB, is highly toxic, with unrepairable ability and strong replication inhibition capacity. When a large amount of clustered DNA damage is produced at once by high doses of radiation, acute cytotoxicity is likely to occur. In most of our studies, we have typically experimented with doses at which a lot of damage occurs [3, 4]. Conversely, only a small amount of DNA damage occurs at low doses. Hence, the indirect effects of mutagenesis are likely more important than the direct cellular effects of damage, such as replication inhibition.

We previously investigated DNA damage on pUC19 plasmid DNA irradiated by gamma-rays and carbon ion beams [3]. The number of DNA lesions usually increases in a dose-dependent manner. On the other hand, we cannot observe the mutagenesis derived from DNA damage in a complete *in vitro* experimental system. This is because mutation requires replication to work against DNA damage. Of course, it is possible to observe the insertion of a base that is not supposed to be inserted as a counterpart of the damaged base in an *in vitro* DNA synthesis system in which purified DNA replication enzymes are loaded, and we have performed many such experiments [3,6–8]. However, it is known that *in vivo*, most DNA damage is eliminated by repair systems, and the frequency of mutations caused by damage is extremely low. Therefore, it is necessary to use a cellular or individual system to observe mutations in response to certain DNA damage. This study investigated the biological effects of heavy ion irradiation via mutation. We conducted irradiation experiments using plasmid DNA as the target molecule and analyzed the mutations converted in the *Escherichia coli* cells. Analysis of DNA damage caused by radiation, including heavy particle radiation, requires relatively high doses of 100 Gy or more due to analytical sensitivity issues, in which case it is challenging to observe milder effects originating from mutations. As radiation-induced DNA damage is a major DSB and directly results in cell death, it is difficult to observe milder effects derived from mutations. We have previously analyzed radiation DNA damage, which required a high dose range. In the present study, we use the low dose range to reveal the pathways of radiation biological effects via mutations, targeting point mutations, and other relatively small mutations in the lower range than the directly observed dose for damage below 100 Gy. Here, we used carbon ions as heavy ion beams. As a result, the mutation spectrum of carbon ion beams was different from that of gamma-rays, which was used as a comparison. Carbon ion beams mainly induce deletion-type mutations, unlike gamma-rays. Base substitutions occurred with both radiations, but each showed a different mutation spectrum. This indicates that the types of base lesions induced by carbon ion beams and gamma-rays may differ, which was investigated using mass spectrometry in this study. The results of this study suggest that carbon ion beams, unlike gamma-rays, induce deletion-type mutations more efficiently and have a higher probability of causing replication inhibition than gamma-rays, even at low doses where clustered DNA damage has a lower yield. In conclusion, carbon ion beams exhibit high biological effectiveness by producing lethal damage more efficiently at low and high doses.

## MATERIALS AND METHODS

### Materials

pUC19 plasmid DNA (2686 bp) (Nippon Gene, Tokyo, Japan) was amplified in the competent cells of *E. coli* HB101 and purified by a QIAGEN Plasmid Mega Kit (Qiagen, Hilden, Germany) as described previously [9]. The competent cells of *E. coli* HB101 were prepared using the method described by Sambrook and Russell with the laboratory stock of the cells [3]. The purified plasmids were primarily of Type I supercoiled form, over 95% (data not shown). Agarose S (Nippon Gene, Tokyo, Japan) was used for agarose gel electrophoresis. The ECOS™ competent cells of *E. coli* JM109 (Nippon Gene, Tokyo, Japan) were used to clone the irradiated plasmid molecules for sequencing. Tryptone, yeast extract and agar were from Becton, Dickinson and Company (Sparks, MD, USA) for bacterial culture. The sequence primers were constructed by Tsukuba Oligo Service (Ushiku, Japan). 8-hydroxy-2′-deoxyguanosine (8-OH-dG) was from Fujifilm Wako Pure Chemical Corp. (Osaka, Japan). 5-hydroxy-2′-deoxycytidine (5-OH-dC) was purchased from Carbosynth (Compton, UK). ^15^N_5_-labeled 8-hydroxy-2′-deoxyguanosine (L-8-OH-dG) and ^13^C, ^15^N_2_-labeled 5-hydroxy-2′-deoxycytidine (L-5-OH-dC) were obtained from Cambridge Isotope Laboratories (Tewksbury, MA, USA) and Toronto Research Chemicals Inc. (Toronto, ON, Canada), respectively. Unless otherwise stated, the other chemicals used in this study were from Fujifilm Wako Pure Chemical Corp.

### Irradiation of DNA

pUC19 DNA was dissolved in 10 mM Tris-HCl (pH 7.5) containing 1M dimethyl sulfoxide (DMSO) to 400 μg ml^−1^ as a concentration. Adding 1M DMSO to the solvent is a common technique to mimic intracellular radical scavenging conditions. In this study, irradiation experiments were carried out by adding 1M DMSO, following the method of Hirayama *et al*. [10] The plasmid DNA solution was irradiated by ^137^Cs-gamma-rays or accelerated carbon ion beams (290 MeV amu^−1^) at room temperature under aerobic conditions. The LET values were 0.9 and 13 KeV μm^−1^ for the gamma-rays and carbon ion beams, respectively. The dose rates were 0.8 and 4 Gy min^−1^, respectively. The gamma-irradiation was performed by a ^137^Cs-gamma source (Atomic Energy of Canada, Chalk River, ON, Canada) in the Analytical Research Center for Experimental Sciences, Saga University, Japan. Carbon ion beam irradiation was done at the Heavy Ion Medical Accelerator in Chiba (HIMAC) at the Quantum Life and Medical Science Directorate, the National Institutes for Quantum Science and Technology, Japan. The irradiated samples were promptly collected and stored at −80°C until analysis. The plasmid DNA was quality-controlled by agarose gel electrophoresis and absorption spectra for further processing.

### Agarose gel electrophoresis

For discussion of mutagenesis induced by DNA base lesion production, it is necessary to compare doses at the same damage level for different radiation types used in this study. For this purpose, irradiated plasmid DNA was subjected to electrophoresis to compare the degree of introduction of DNA strand breaks with radiation DNA damage. A portion of the irradiated DNA sample (50 ng) was added to a gel loading buffer consisting of 0.25% (w/v) bromophenol blue, 30% (w/w) glycerol, and 1M ethylenediaminetetraacetic acid disodium salt dihydrate (EDTA). Then, the sample was separated on a standard 0.8% (w/v) agarose gel prestained with ethidium bromide (100 ng ml^−1^) in TAE buffer consisting of 40 mM Tris, 40 mM acetic acid, and 1 mM EDTA at 50 V for 60 min. The gel was then imaged on an E-Graph (ATTO, Tokyo, Japan), and the Type I-III bands were quantified by Image J software (National Institute of Health, Bethesda, MD, USA) [11]. The strand breaks as DNA damage in the plasmid irradiated by ionizing radiation is analyzed by the conformational changes on agarose gel electrophoresis. Supercoiled plasmid DNA (Type I) is transformed into open circular (Type II) by the generation of a single-strand break (SSB) and into linear (Type III) by the generation of a DSB, respectively [3].

### DNA cloning and sequencing

According to the instructions, the respective irradiated DNA was transformed into competent cells. The competent cells were thawed on ice, and 1 ng of the irradiated DNA was mixed and kept on ice for 5 min. The mixture was at 42°C for 45 s and then seeded on a LB agar plate containing ampicillin, 5-bromo-4-chloro-3-indolyl-β-D-galactopyranoside (X-gal) and isopropyl-β-D-thiogalactopyranoside (IPTG). The LB medium with the additives consisted of these components: 1% (w/v) tryptone, 0.5% (w/v) yeast extract, 1% (w/v) NaCl, 1.5% (w/v) agar, 50 μg ml^−1^ of ampicillin, 40 μg ml^−1^ of X-gal and 200 μg ml^−1^ of IPTG. The inoculated plates were incubated at 37°C. After one day of incubation, the white colonies harboring mutations in the lacZα region of the plasmid were collected. The pUC19 plasmid encodes a lacZα gene, and the *E. coli* JM109 has lacZΔM15. Both lacZα and lacZΔM15 are co-expressed, and the whole activity of the lacZ gene product appears, which is called α-complementation [12]. In this system, IPTG induces the corresponding gene expression, and the gene product digests X-gal into a blue-colored product, forming blue colonies. Therefore, lacZα gene inactivation with base alterations results in the appearance of a white colony. Then, the respective mini-scale liquid cultivation of selected white clones (in 2 ml of LB liquid medium) was done to recover the plasmid DNA. Extraction and purification of the plasmid DNA with mutations were done using the QIAprep Spin Miniprep Kit (Qiagen). The purified plasmid DNA was quality-controlled by agarose gel electrophoresis and absorption spectra and taken to the sequencing process. The lacZα sequences were analyzed with an Applied Biosystems 3130xl Genetic Analyzer (Waltham, MA, USA) using BigDye Terminator v3.1 Cycle Sequencing Kit (Applied Biosystems). The sequencing primers were 5′-GCT TGT CTG TAA GCG GAT GC-3′ and 5′-GCG GGC AGT GAG CGC AAC GC-3′, as forward and reverse ones, respectively. These sequencing primers are used so frequently for sequencing lacZα regions that they are commercially available. As shown in the unirradiated section of Table 1, the sequencing of the lacZα region before irradiation was checked to ensure that no spontaneous mutations were included.

**Table 1:** Mutation spectra in lacZα region of pUC19 DNA irradiated by gamma-rays and carbon ion beams

Radiation			Gamma-rays		Carbon ion beams	
Dose (Gy)		0	25	50	25	50
Survival rate (%)		100.0	32.5	9.9	33.6	2.0
Mutation frequency (%)^*^		0.5	0.8	3.2	7.4	29.5
Transition	G:C ↔ A:T	0	1	0	3	6
Transversion	G:C ↔ T:A	0	1	7	2	1
	G:C ↔ C:G	0	1	5	0	0
Deletion	Δ1	0	0	1	8	2
	Δ2	0	0	0	1	0
Mutation summary		0	3	13	14	9

^*^Mutation frequencies are calculated from the blue/white selection result for colonies of clones harboring irradiated pUC19. The number of sequence analyses in each series was at least 47.

### Mass spectrometry

The irradiated plasmid DNA was hydrolyzed by 3 U of nuclease P1 in a 100 μl solution at 37°C for 60 min, and then we obtained the relevant nucleotide components. The nucleotide mixture was next digested with 1.2 U of alkaline phosphatase in 10 mM Tris-HCl (pH 8.5) at 37°C for 60 min to yield the nucleosides. L-8-OH-dG and dL-5-OH-dC reagents were added to the nucleoside mixture as an internal standard. Then, the samples were passed through a Nanosep Filter Centrifugal Device (Pall, Port Washington, NY, USA) to remove any debris that may damage the chromatography column. Mass spectrometric (MS) analysis of oxidative base lesions was performed by liquid chromatography coupled with tandem mass spectrometry (LC–MS/MS) using an LCMS-8030 instrument (Shimadzu, Kyoto, Japan), as reported previously [9]. The nucleoside mixtures were separated at 40°C through a KINETEX 1.7 μm C18 column (ϕ 2.1 mm × 100 mm) (Phenomenex, Torrance, CA, USA) in the isocratic mobile phase of 0.1% (v/v) aqueous formic acid containing 5% (v/v) acetonitrile. MS data acquisition was performed in multiple-reaction monitoring (MRM) mode. For 8-OH-dG with L-8-OH-dG, the collision energies were optimized at 15 and 16 eV, and the MRM transitions were from 284 to 168 m/z and from 289 to 173 m/z, corresponding to the precursor and the product ions, respectively. For 5-OH-dC with L-5-OH-dC, the collision energies were optimized at 12 and 13 eV, and the MRM transitions were from 244 to 128 m/z and from 247 to 131 m/z, corresponding to the precursor and the product ions, respectively.

## RESULTS

**Fig. 1:**
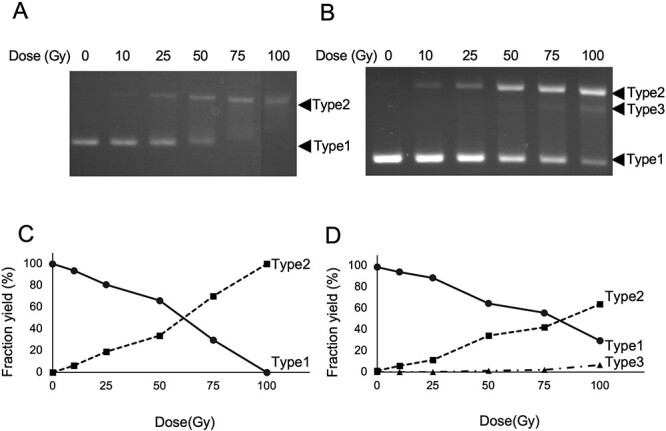
The typical images of agarose gel electrophorese for pUC19 DNA irradiated by gamma-rays (A) and carbon ion beams (B). Plots of the yields of Types I, II and III of pUC19 DNA irradiated by gamma-rays (C) and carbon ion beams (D) for the doses from the gel image analyzed by Image J. Respective plots were from the average of two independent experiments.

Ionizing radiation induces DNA damage dose-dependently, but too much DNA damage inhibits DNA replication and makes it impossible to induce mutations. This means that the dose at which mutations occur with high efficiency must be determined to observe radiation-induced mutations. In addition, the gamma-rays and carbon ion beams compared in this study have different LET and different relative biological effectiveness (RBE), so the respective doses that introduce the same level of damage must be determined. Therefore, we first irradiated pUC19 DNA with gamma-rays or carbon ion beams up to 100 Gy and analyzed the damaging generation by agarose gel electrophoresis. As described in Materials and Methods, agarose gel electrophoresis under certain conditions can separate SSB and DSB occurring on circular plasmid DNA, such as pUC19, as distinct bands [3]. Gamma radiation dose-dependently increased type II in the range up to 100 Gy and correspondingly decreased type I (Fig. 1A and C). Type I shows the development of intact cyclic plasmid DNA, and type II shows SSBs. Over the same dose range, carbon ion beams also dose-dependently increased type II and decreased type I (Fig. 1B and D). Carbon ion beams also produced type III, which gamma-rays could not observe. To compare the mutations between gamma-rays and carbon ion beams, it was preferable to compare them at the same damage level rather than at the same dose. That is because many previous studies, including ours, have reported different results for heavy ion particles and gamma-rays in terms of damage yield [5], and because too high a damage level would make it difficult to detect mutations due to replication inhibition, we chose a dose that left a relatively intact fraction. There was no significant difference in the yield of strand breaks between gamma and carbon-ion radiations in the dose range considered in this study (Fig. 1). The yields of Type I and II for 25 Gy of gamma-rays were 80.9% and 19.1%, respectively. Those were 88.7% and 11.3% for the same dose of carbon ion beams. The yields of Type I and II for 50 Gy of gamma-rays were 66.2% and 33.8%, respectively, and those were 64.6% and 34.2% for the same dose of carbon ion beams. Additionally, 50 Gy of carbon ion beams induced 1.2% of Type III, which was detected for the first time. As a result, the study chose to detect mutations at 25 and 50 Gy with gamma-rays and carbon ion beams.

The blue/white selection of clones harboring irradiated pUC19 DNA indicated the mutation in the lacZα region. The white colonies that were most likely to harbor the mutation were selected, and after liquid culture, each plasmid DNA was extracted, purified, and sequenced for the lacZα region. Table 1 summarizes the results of these mutation analyses. The survival rate, calculated as the colony formation rate per unit weight of plasmid DNA, decreased with dose elevation, and the rates with carbon ion beams were uniformly lower than those with gamma-rays. This suggests that ionizing radiation harms the extracellular DNA in a dose-dependent manner, reaching levels at which its replication in the host cell ultimately becomes impossible at high doses. The damaging ability of carbon ion beams is higher than that of gamma-rays [3, 4]. As shown in Table 1, 25 Gy of gamma irradiation resulted in very low yield mutagenesis. The mutations were all base substitutions. Gamma-rays also increased the number of mutations at 50 Gy. Most of these were transversions involving guanine (G). G:C↔T:A and C:G↔A:T transversions were prominent after 50 Gy of gamma irradiation. On the other hand, mutation production by carbon ion irradiation showed a different aspect from that by gamma irradiation. The significant mutations the carbon ion beams induce are C:G↔T:A transitions and single base deletions (Table 1). This difference in mutations induced by gamma-rays and carbon ion beams may be due to differences in the DNA damage produced by the respective radiations, as discussed later. This is consistent with the fact that the mutations in this study that may be attributed to 8-oxoguanine (8-OH-G) are rarely seen with carbon ion beams, but only with gamma-rays (Table 1).

**Fig. 2:**
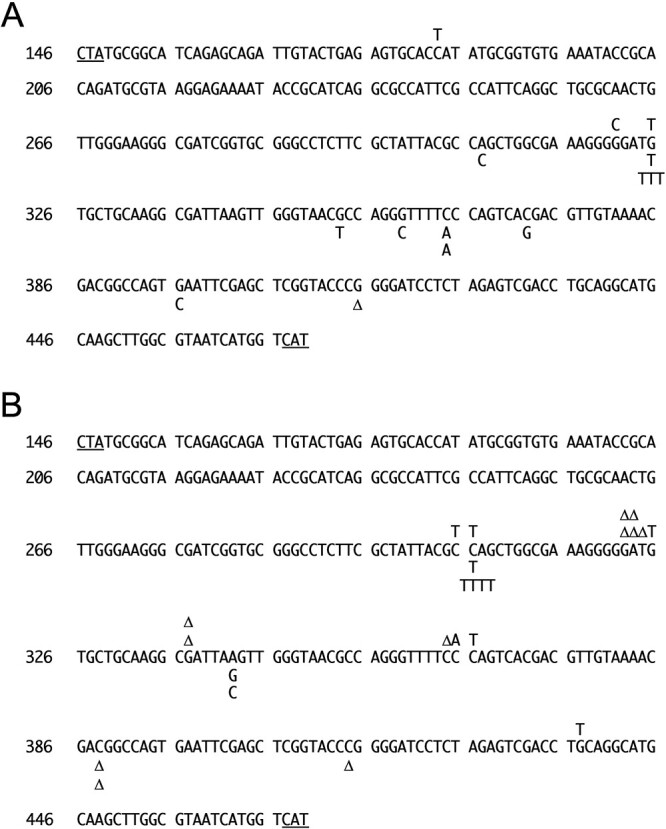
Mutation map of the lacZα regions of pUC19 DNA irradiated by 25 and 50 Gy of gamma-rays (A) and carbon ion beams (B). We performed sequencing on the entire lacZα ORF, which is represented in this figure. As the numbering of Genbank indicates this sequence: M77789.2, this ORF is shown reverse and complemented. The start and stop codons are underlined at CAT and CTA at the tail and leading end of the sequence, respectively. The 25 and 50 Gy mutations are shown at the top and bottom of the sequence in respective figures. The numbers to the left of the sequence are the official numbers for the pUC19 sequence in the nucleotide sequence database.

These mutations are differentially distributed on DNA by gamma-rays and carbon ion beams (Fig. 2). For gamma-rays, G_325_ is a hot spot for G:C↔T:A transversion. The very close upstream of G_325_ is a sequence of the G-run (G_318_–G_322_). Interestingly, the same G-run (G_318_–G_322_) appears to induce different mutations with gamma-rays and carbon ion beams, as discussed later. Other mutation sites at G_309_, G_353_, C_364_, C_372_ and G_396_ caused by gamma-rays are sporadic, except for the other two sites (Fig. 2A). One of the two different sites for gamma-rays is C_183_, showing the C:G↔T:A transition. Another one for gamma-rays is G_415_, which shows a deletion. This site is in a G run (G_415_–G_418_).

For carbon ion beams, the distribution of mutations showed a different picture for gamma-rays. Of course, the mutation spectrum is different between gamma-rays and carbon ion beams, as mentioned earlier, but their production locations are also different. Suppose regions showing three or more cases of mutation are interpreted as hotspots. In that case, three locations are relevant in the result with carbon ion irradiation: CC_305–306_, GATG_322–325_ and CCC_364–366_ (Fig. 2B). CC_305–306_ shows all C:G↔T:A transitions. GATG_322–325_ shares its location with hotspots observed during gamma irradiation as G_325_, although the type of mutation is different. For carbon ion beams, GATG_322–325_ shows almost all deletions except G_325_, showing G:C↔T:A transversion. The last hotspot is CCC_364–366_, showing various mutations that are thought to be involved in charge migration with opposed G runs. The rest of the mutations are sporadic, but one of them, C_364_, is in a C run (CCC_364–366_), and there is a G run (GGGG_365–368_) in the neighborhood, so it might be due to charge transfer with GGGG_365–368_ or opposed GGG_364–366_.

**Fig. 3:**
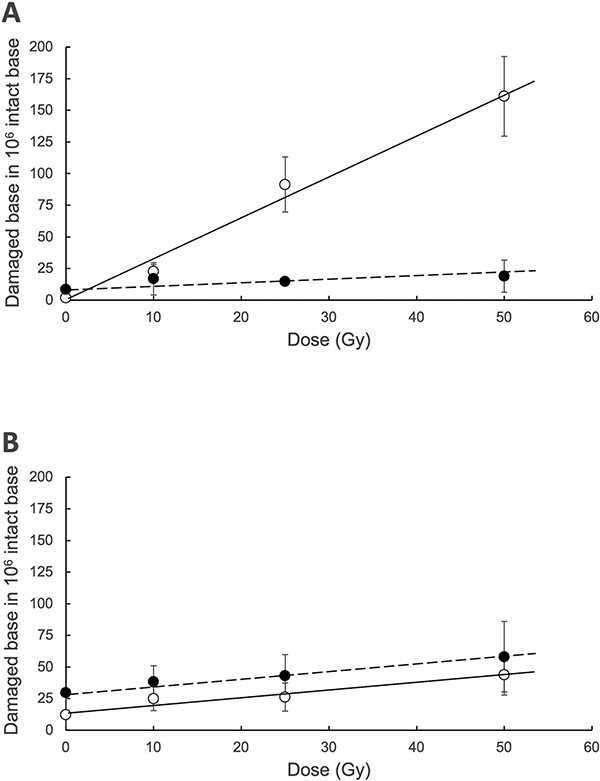
Yields of 8-OH-G and 5-HO-C in pUC19 DNA determined by LC–MS/MS for dose of gamma-rays (A) and carbon ion beams (B). The horizontal axis shows the dose (Gy), and the vertical axis shows the damaged bases (8-OH-G or 5-HO-C) to 10^6^ of G or C in DNA. Open circles and straight lines indicate 8-OH-G, and closed circles and dotted lines indicate 5-HO-C. The results represent the mean of at least three experiments with their standard deviations. If the standard deviation is small, the line indicating it may be hidden from view in the plot.

To explore the causes of the differences in mutagenesis between gamma-rays and carbon ion beams, the yields of the damaged bases 8-OH-G and 5-hydroxycytosine (5-HO-C), which are thought to be responsible for the major mutations in each, were observed. The DNA irradiated with the respective radiations was digested into nucleosides and subjected to mass spectrometry to determine the amount of the two oxidized bases produced. The production of 8-OH-G was overwhelmingly higher in gamma-rays than that of 5-HO-C (Fig. 3A). Carbon ion beams did not produce large amounts of 8-OH-G unlike gamma-rays. The slopes of the production curves of 8-OH-G and 5-HO-C by carbon ion beams were almost identical (Fig. 3B). The background is higher for both damages in carbon ion beams, especially for 5-HO-C compared to 8-OH-G, than in gamma-rays. This may be due to the slightly more time-consuming process involved in carbon ion beam irradiation in HIMAC than gamma irradiation.

## DISCUSSION

When high doses of ionizing radiation produce large amounts of DNA damage in a cell that exceed the DNA repair capacity, but most of the damage is not removed, the cell is expected to die from direct inhibition of replication rather than from the mutational effects it produces. The biological effects of radiation can be divided into direct cell death at high doses and indirect cell death via mutation at low doses, and we have already reported an analysis of the former produced by heavy particle radiation, including carbon ion beams, using high doses. In the present study, we wanted to analyze the latter, which remains to be done. When a DNA molecule is irradiated too much, even if introduced into the cell, it will lose its replication ability, and it may not be possible to observe the mutational potential of the molecule. Thus, what we needed to do was first determine the dose at which mutations could be observed. In addition, mutagenesis is not linear with dose and often reaches a maximum at a certain dose. This is because as cell death increases with dose for the reasons described above, the number of cells showing mutations decreases as the number of living cells in the denominator decreases. This is another reason for determining the experimental dose. Therefore, the first experiment in this study was to determine the dose at which the mutations would be observed. We decided to carry out irradiation experiments at 25 and 50 Gy for both gamma-rays and carbon ion beams (Fig. 1). In this study, DNA strand breaks were used as an indicator for determining the dose at which mutations are analyzed. DNA strand breaks, including DSBs, are radiation-specific and replication-inhibiting damages that act in an inhibitory manner on the expression of point mutations. Therefore, it was necessary to search for dose regions where these damages are somewhat less and point mutations are more likely to be expressed. The higher RBE of carbon ion radiation compared to gamma radiation led to the expectation of a higher damage yield in the latter. However, in practice, there was no significant difference in the yield of chain breaks between the two in the dose range up to 50 Gy, so we decided to compare the two at the same dose. Although there is controversy about using strand breaks as an indicator for point mutation detection, it came out better for point mutation detection (Table 1).

The mutation spectra obtained in this study are summarized in Table 1. For gamma-rays, the mutations were all base substitutions except one. Most of these were transversions involving guanine (G). G:C↔T:A and C:G↔A:T transversions were prominent after 50 Gy of gamma irradiation. This may be due to oxidative damage of guanine, 8-OH-G [13, 14]. This result is consistent with the fact that gamma-rays, which have a high proportion of indirect effects, have a high yield of 8-OH-G. G-oxidation *in vivo* occurs at two locations: on chromosomal DNA and in the nucleotide pool. In the former case, chromosomal G is converted to 8-OH-G, which is used as a template for the first replication to insert adenine (A) as the complementary base, and thymine (T) is inserted at the position of 8-OH-G as a template to complete the mutation [15, 16]. In the latter case, 8-OH-G in the deoxyribonucleotide triphosphate form is inserted into the chromosomal A, and cytosine (C) is inserted at the position of A using it as a template, thus completing the mutation [17–19]. In this experiment, only the former event occurs since oxidation in the nucleotide pool is out of scope. The next most common mutation in gamma-rays is G:C↔C:G transversion. This mutation is one of those caused by active oxidative treatments, including irradiation, and is thought to be induced by 8-OH-G [20–23].

Unlike gamma-rays, the mutation production by carbon ion beams showed a different aspect. The significant mutations the carbon ion beams induce are C:G↔T:A and single base deletions (Table 1). The former mutation is thought to be derived from 5-HO-C, an oxidative damage of cytosine [24, 25]. 5-HO-C is known to cause the C:G↔T:A transition, and the result of this study suggests the involvement of 5-HO-C [26–29]. 5-HO-C may be involved in yet another mutation. Several of the previously mentioned references report that 5-HO-C induces C:G↔G:C transversions [26, 27]. 5-HO-C may be involved in some of the C:G↔G:C transversions in this study. Interestingly, the mutation that appears to be derived from 8-OH-G is primary in gamma-rays, while the mutation that seems to be derived from 5-HO-C is primary in carbon ion beams. 8-OH-G has been reported to decrease the formation yield in a LET-dependent manner [30]. There are many reports of similar LET-dependent reductions in DNA damage yields, including ours [3–5, 31, 32]. In many of these studies, both isolated and clustered DNA damage reduced yields in a LET-dependent manner. The decrease in 8-OH-G yield is more pronounced under oxic conditions and is rarely seen under hypoxic conditions [30]. This is consistent with the fact that the mutations in this study that may be attributed to 8-OH-G are rarely seen with carbon ion beams but only with gamma-rays (Table 1). The LET-dependent decrease in DNA damage yield is due to a localized increase in the density of H_2_O-derived primary active product formation in a LET-dependent manner and its lower yield due to recombination reactions [33, 34]. The LET-dependent decrease in DNA damage yield is more pronounced for isolated ones and only slightly reduced for clustered ones [3–5, 31, 32]. This suggests that the effect of cluster DNA damage is more pronounced at high LET radiation, which is consistent with the fact that single and double nucleotide deletions are major with carbon ion beams in this study (Table 1). Also, this suggests that carbon ions produce damage that inhibits the progress of DNA replication enzymes. Such damage would be clustered DNA damage, including DSB. We have also confirmed that clustered DNA damage is hardly repaired and has high replication inhibition potential [3, 4, 35]. In the case of carbon ion irradiation, unlike gamma irradiation, the number of mutations decreased with increasing doses from 25 to 50 Gy. At 50 Gy, the blue/white selection mutation rate was 29.5%. However, the survival rate obtained from the transformation efficiency was low, at 2.0%. It is suggested that 50 Gy of carbon ion irradiation causes damage that expresses mutations and a lot of damage, making it difficult for the plasmid to replicate. Of course, one of the DNA damages is DSB, as known. DSB is damage that exhibits extremely high DNA replication inhibitory capacity and induces mutations with high frequency [1, 2, 5, 35, 36]. Phillips and Morgan reported that a deletion of 1–36 bp is caused by DSB, which was modeled as one made by restriction enzymes [35]. Conversely, deletions have also been found to occur in clustered DNA damage other than DSB. Shikazono *et al*. [37] showed DNA replication using artificially created clustered base lesions as templates and reported that clustered base lesions induce deletions. Thus, our result here is consistent with these previous studies.

The distribution of mutations also depends on the type of radiation (Fig. 2). For gamma-rays, G_325_ is a hot spot for G:C↔T:A transversion. It is well known that G:C↔T:A transversion is derived from G-oxidation generating 8-OH-G [15, 18]. The very close upstream of G_325_ is a sequence of the G-run (G_318_–G_322_). It is well known that charges (cation radicals) generated on DNA oxidized by ionizing radiation can migrate up the DNA strand to G, which has the lowest oxidation potential, eventually establishing that G is an oxidative damaging target [38, 39]. This G-run (G_318_–G_322_) induces different mutations with gamma-rays and carbon ion beams. Other sporadic mutation sites at G_309_, G_353_, C_364_, C_372_ and G_396_ caused by gamma-rays are mostly translocations thought to originate from 8-OH-G (Fig. 2A). One of the two other sites for gamma-rays is C_183_, which might be involved in 5-HO-C, an oxidative C. 5-OH-C can pair not only G but also A, as mentioned above [26–29]. G_415_ deletion with gamma-rays is in a G run (G_415_–G_418_), and the mutation might also be derived from charge migration or replication slippage.

On the other hand, CC_305–306_ shows all C:G↔T:A transitions. This might be derived from C oxidation leading to 5-HO-C with carbon ion irradiation [26–29]. GATG_322–325_, showing almost all deletions, is thought to be involved in charge migration with the combination of the G run (G_318_–G_322_) and replication slippage for carbon ion beams.

The results of mass spectrometry analysis of DNA base lesions showed differences in mutagenesis by gamma-rays and carbon ion beams: the yield of 8-OH-G was much higher than the yield of 5-HO-C by gamma radiation (Fig. 3A). This is consistent with the result that the significant mutation induced by gamma rays was a G:C↔T:A rearrangement, which is thought to originate from 8-OH-G (Table 1). In contrast, the production curves for 8-OH-G and 5-HO-C were almost identical in the carbon ion beams (Fig. 3B). This is consistent with the fact that few 8-OH-G-derived mutations were observed in the carbon ion beams (Table 1); the slightly higher yield of 5-HO-C compared to 8-OH-G is consistent with the finding of possible 5-HO-C-derived mutations in the carbon ion beams. These results indicate that the mutations induced by gamma-rays and carbon ion beams are different due to the DNA damage produced by each type of radiation. Furthermore, although somewhat wildly calculated, the total amount of damage in 8-OH-G and 5-HO-C combined is lower in carbon ion beams than in gamma-rays. Still, the total number of mutations is conversely higher in the latter. This suggests that the point mutations in carbon ion beams may not originate solely from isolated base lesions but also from lesions that cannot be quantified by mass spectrometry, mainly clustered DNA lesions, including DSBs. This is because the mutations with carbon ion beams are biased toward deletions, which recalls the presence of replication-inhibiting damage.

In summary, this study has allowed us to identify the differences in mutation trends between gamma-rays and carbon ion beams and the damage that contributes to these differences. Of course, the study needed to provide a complete picture of mutations, as it only looked at mutations in a limited area of the irradiated samples. For example, the analysis in this study was limited to the open reading frame (ORF) of lacZα of pUC19 plasmid DNA, and the analysis of its regulatory regions and other regions related to plasmid survival was not possible. In addition, even though only two primary base lesions were considered as the cause of mutations in this study, and most of the mutations detected could be explained, it is known that many oxidative base lesions can be caused by radiation, and their effects should naturally be considered. However, we believe that this objective will be achieved by future developments based on the results of this study.
